# Declining Condom Use Among Sex Workers in Western Australia

**DOI:** 10.3389/fpubh.2018.00342

**Published:** 2018-11-27

**Authors:** Linda A. Selvey, Jonathan Hallett, Kahlia McCausland, Julie Bates, Basil Donovan, Roanna Lobo

**Affiliations:** ^1^School of Public Health, The University of Queensland, Herston, QLD, Australia; ^2^Collaboration for Evidence, Research and Impact in Public Health, School of Public Health, Curtin University, Bentley, WA, Australia; ^3^Urban Realists Planning & Health Consultants, Sydney, NSW, Australia; ^4^The Kirby Institute, University of New South Wales, Sydney, NSW, Australia

**Keywords:** sex work, condoms, sexually transmissible infections, Australia, natural sex

## Abstract

**Introduction:** Since the emergence of the HIV pandemic in the 1980s, high rates of condom use for penetrative sex have been reported among sex workers in Australia. The sex industry in Australia and elsewhere has changed over the previous decade with increasing proportions of sex workers working privately and lower proportions of sex workers working in brothels. There has also been some evidence of reductions in condom use, particularly during penetrative oral sex. We therefore decided to investigate sex workers' use of condoms during penetrative sex with clients.

**Methods:** This was a cross-sectional mixed methods study of sex workers in Western Australia. The study involved an environmental scan of the sex industry in Western Australia, visits to brothels and other sexual services premises, a survey of sex workers and in-depth interviews.

**Results:** We surveyed 354 male, female, and transgender sex workers in Western Australia. We found unexpectedly low rates of reported condom use with clients for all forms of penetrative sex. Of respondents who reported providing this type of service, 33% reported that all clients used condoms during oral sex, 69% during vaginal sex and 59% during anal sex. High levels of client demand for condomless sex was also reported, with 42% of sex workers reporting that all or most clients requested condomless oral sex. Increasing client demand, fear of losing clients and the ability to charge more for condomless sex were reported reasons for providing this service.

**Conclusions:** There has been an apparent increase in sex workers reporting condomless penetrative sex with clients in Western Australia compared to a previous cross-sectional study. An increase in private sex work and client demand for condomless sex together with an economic downturn leading to increased competition for clients may be important contributing factors.

## Introduction

In response to the emerging human immunodeficiency virus (HIV) pandemic in Australia in the 1980s, Australian sex workers instituted high rates of condom use with clients and regular sexual health checks that resulted in rates of HIV and sexually transmissible infections (STI) among sex workers that were equivalent to or even lower than in the general population (1). A study undertaken in 2007 found that 100% of female Perth brothel-based sex workers who provided vaginal sex to clients and 96% who provided oral sex to clients reported condoms being used all of the time (2). A similar study in Sydney in 2007 found that 94% of female brothel-based sex workers who provided vaginal sex to clients and 84% of those who provided oral sex to clients reported condoms being used 100% of the time (3). Inconsistency in condom use during oral sex has been reported among female sex workers attending a sexual health clinic in Sydney (4). In this study, around 25% of respondents reported condoms being used inconsistently during oral sex, although the majority of respondents reporting condoms being used inconsistently during oral sex said that condoms were still used at least 80% of the time (4).

The use of condoms during sex work has been reinforced and supported over time by outreach, peer education and support by community-based organizations around Australia (5). Peer-based sex worker organizations exist in most Australian jurisdictions, and these are members of a national sex worker organization, Scarlet Alliance (6). Some sexual services premises display signs reminding clients of the need to use condoms and most Australian states and territories have legislation that makes condomless sex with a sex worker illegal: Western Australia (WA Prostitution Act 2000), Australian Capital Territory (ACT Prostitution Act 1992), Victoria (Sex Work Act 1994), Queensland (Prostitution Act 1999), and Northern Territory (NT Prostitution Regulation Act 2004). In Western Australia (WA) there were small numbers (10 per year) of prosecutions for “failure to use a prophylactic” in the early 2000s. In contrast there was one prosecution for this offense in the years 2011–2015 (7).

There are three different legislative approaches to sex work in Australia: decriminalization, regulation, and criminalization (7). In WA private sex work is not an offense. However, street-based sex work is illegal and it is illegal to live off the earnings of another's sex work. Brothels are also illegal (7). There is no evidence that criminalization results in a reduction in sex work (8). Decriminalization on the other hand, may result in better health outcomes for sex workers (2, 9). There have not been any changes in WA legislation in relation to sex work between 2007 (when the previous study was undertaken) and the present. Between 2007 and 2012, the WA economy boomed, and peaked in 2012. Following the peak it declined back to 2007 levels (10).

Very little is documented about the size and composition of the sex industry in Australia. For example, prior research in New South Wales using a variety of methods estimated the number of sex workers in that state to be anywhere between 1,500 and 10,000 (3). However, many have observed that in the last decade the sex industry in Australia and other countries has changed with an increasing proportion of private sex workers working outside of brothels. In Australia, increased numbers of sex workers from Asian countries (particularly China, Korea and Thailand) have also been observed (11). Therefore, we considered it to be timely to investigate the health and safety of sex workers in WA (7). This was a broad study that addressed a range of objectives including those relating to condom use with clients. The objectives of the aspects of the study reported here were to assess the proportion of sex workers reporting that clients requested and participated in condomless sex, and to investigate the factors associated with condomless sex with clients.

## Materials and methods

This was a cross-sectional mixed methods study of the sex industry in WA, involving perusal of advertisements, a survey of sex workers, visits to sexual services premises and in-depth interviews with sex workers. For the purposes of this study we defined a sex worker as someone who provides sexual services (sexual intercourse with, or masturbation of another person, using any part of the body or an object) in exchange for financial gain.

### Ethics

The study was approved by Curtin University Human Research Ethics Committee, approval number HRE2016-0078. All participants provided informed consent to participate according to the Declaration of Helsinki. Following perusal of an information statement, participants ticked a box on the online survey indicating consent. For interviews, participants ticked a box on a paper survey indicating consent that was countersigned by the investigator. This was done to protect the participants' anonymity.

### Peer researchers

Eight current or former sex workers and a brothel receptionist were recruited as peer researchers and were trained by an investigator (JB), who is also a peer. The peer researchers undertook visits to sexual services premises including brothels, and were involved in recruitment of study participants. JB was involved throughout the study from its development to reviewing study findings and contributing to manuscripts. Additional peers, who were representatives of peer-based sex worker organizations, also contributed to the study design and reviewed our study findings.

### Perusal of advertisements

The Google search engine was used to search the following search terms–escort, massage, brothel, sex, parlor, sensual, sexual, services, and classified. These search terms were identified from the literature and from discussions with peers. In addition, a number of online advertising sites known to advertise sexual services were perused. Advertisements from local newspapers throughout WA were also perused. These advertisements were perused to obtain contact details to promote our online survey. Any advertisements that were found to offer condomless (or natural) sex were noted.

### Visits to brothels and other sexual services premises

We developed a list of sexual services premises and their locations after scanning the advertisements. This list was augmented by peers and our peer researchers. We compiled two separate lists: those with predominantly English-speaking sex workers and those with predominantly Asian sex workers. We removed a number of premises from the initial list after being informed by peer researchers that they no longer existed or could not be found. For contacting and visits, peer researchers were provided with a random selection of Perth-based brothels with a majority of English-speaking workers offering full service (penetrative sex). Where a premises was found to be closed or the owner/operator refused a visit, an additional premises was selected. Attempts were made to visit all premises identified as providing full service by predominantly Asian sex workers, as we expected that a number of such premises would not be identified. To increase the number of premises with predominantly Asian sex workers visited, attempts were made to also visit a number of massage shop front premises that were identified by peer researchers.

Peer researchers undertook all visits to sexual services premises. The primary reason for the visits to the sexual services premises was to recruit sex workers to participate in the study. During these visits peer researchers also undertook a venue audit using a structured audit tool. This audit tool included the presence of signage stating a requirement to use condoms during penetrative sex, as well as other infrastructure and resources aimed at improving occupational health and safety of sex workers, such as a lunch room, duress alarms and security cameras.

### Sex worker survey and survey recruitment

The sex worker survey instrument was based on a survey that was used previously (2). Additional questions were included in the survey following consultation with key stakeholders including peer-based sex worker organizations. The survey was self-administered either online or on paper, unless requested by the respondent when it was completed with the assistance of a peer researcher. The survey included demographic information; questions about access to information and health services; condom usage; drug and alcohol use; interactions with police; and experiences of violence, stigma, and mental health. The question about condom use with clients was: “In an average week, how many of your clients use condoms or other protection for the following services… (oral sex, vaginal sex, anal sex)”. The options for response were “all clients, most clients, some clients, no clients, or I don't have this type of sex with clients.” We did not provide a definition of “most” or “some” clients. The survey also included a validated question about binge drinking (12). The survey included options for open responses for a number of questions. The survey was translated into Korean, Thai and Chinese by NAATI (National Accreditation Authority for Translators and Interpreters) accredited personnel and checked by peers from Scarlet Alliance, the Australian Sex Workers Association, for appropriate use of language and context. These languages were the most common native languages of Asian sex workers in Perth. Participants received AUD30 in cash for a completed face-to-face survey or a gift voucher for a completed online survey.

Survey participants were recruited via social media (Facebook and Twitter); advertisements in print media; notifications to e-lists and sex worker organizations; visits to sexual services premises; text messages to private sex workers identified via perusal of online and newspaper advertisements; promotional materials left at businesses likely to engage or have contact with sex workers; and via personal networks of peer researchers.

### In-depth interviews

Semi-structured in-depth interviews were conducted after the survey, and an interview guide was developed based on themes of interest arising from the survey results. Sex worker survey respondents were invited to indicate an interest in participating in an in-depth interview and other participants were recruited through word of mouth by peers, peer researchers and peer organizations.

The interviews were conducted by four investigators (LS, RL, JH, KM) either in person or over the phone. Interviews lasted between 30 and 90 min and all but two (who declined) were audio-recorded and transcribed.

### Data analysis

Quantitative: Frequency analyses were undertaken of the survey responses and when comparisons were made between groups of respondents, Mantel Haenszel Chi squared analysis was undertaken or where appropriate Fischer's Exact Test to estimate *p* values. Data analysis was undertaken using SPSS v24 (IBM Analytics, New York, USA).

Qualitative: Qualitative responses in languages other than English were translated to English by NAATI accredited personnel. Some qualitative responses to questions in the survey were coded either manually or by searching for sub-strings within the responses.

The semi-structured interview transcripts were analyzed thematically to identify common themes in the data and any emerging themes that warranted further exploration in subsequent interviews as described elsewhere (7). Descriptive codes or labels were assigned to items of interest and related codes were then grouped into categories to develop overarching themes that addressed the research questions (13). Data management and coding was conducted using NVivo v11 (QSR International).

## Results

### Perusal of advertisements

We perused 223 advertisements for sexual services. Of these, 35 (16%) advertised condomless services including 23 (10%) for oral sex only, and nine (4%) for other forms of penetrative sex. Three did not specify the service. We visited 22 sexual service premises. Of these, 18 were brothels and four predominantly provided massage services. Four of the 22 (18%) premises displayed signs in waiting areas indicating to clients that the use of condoms was required.

### Sex worker survey

There were 354 respondents to the survey, of whom 213 (60%) responded online. Eighty one percent of respondents were women. The highest proportion of respondents (36%) were born in Australia, and 15% were born in either China, Hong Kong or Taiwan. Over half of the respondents (52.5%) were aged 30 years or younger and almost one half (46%) had been working in the sex industry for 2 years or less. More than half of the respondents (55%) reported doing at least some private sex work (Table 1).

**Table 1:** Sex worker survey respondent demographics and characteristics.

**Characteristic (number responding to this question)**	**Number**	**Percent**
**SEX ASSIGNED AT BIRTH (354)**		
Male	68	19.2
Female	286	80.8
**CURRENT GENDER IDENTITY (351)**		
Man	58	16.4
Woman	286	80.8
Genderqueer	10	2.8
**AGE GROUP (349)**		
18–25 years	87	25.0
26–30 years	96	27.5
31–40 years	102	29.3
41–50 years	76	13.2
51 plus years	18	5.2
**COUNTRY OF BIRTH (353)**		
Australia	126	35.6
New Zealand	13	3.7
China/Hong Kong/Taiwan	53	15.0
Thailand	29	8.2
Korea	15	4.3
Malaysia	14	4.0
Other	73	21.0
**YEARS WORKED IN THE SEX INDUSTRY (339)**		
< 1 year	50	14.7
1–2 years	107	31.6
3–5 years	90	26.5
6–10 years	53	15.6
>10 years	39	11.5
**CURRENT TYPE OF SEX WORK (COULD SELECT MORE THAN**		
**ONE ANSWER)**		
Private worker	196	55.4
Massage shop	119	33.6
Parlor or brothel	83	23.4
Escort agency	64	18.1
Street-based	24	6.8
Other	20	5.6

A high proportion of respondents (42%) reported that all or most clients requested oral sex without condoms in an average week (Table 2). A lower proportion of Asian respondents (respondents born in an Asian country) reported that all or most clients requested oral sex without condoms (23%) compared to non-Asian respondents (49%; *p* < 0.001). A lower proportion of respondents reported that all or most clients requested condomless vaginal or anal sex compared to oral sex, with <20% of female respondents reporting that all or most clients requested condomless vaginal sex in an average week (Table 2).

**Table 2:** Proportion of respondents reporting that clients requested sex without condoms in an average week.

	**Vaginal sex**	**Anal sex**			**Oral sex**		
	**Women only** *n =* **262** ***n (*%)**	**Men** *n =* **53** ***n (*%)**	**Women** *n =* **256** ***n (*%)**	**All** *n =* **318** ***n (*%)**	**Men** *n =* **54** ***n (*%)**	**Women** *n =* **260** ***n (*%)**	**All** *n =* **323** ***n (*%)**
All clients	18 (6.9)	3 (5.7)	7 (2.7)	11 (3.5)	9 (16.7)	25 (9.6)	34 (10.5)
Most clients	31 (11.8)	7 (13.2)	22 (8.6)	31 (9.8)	20 (37.0)	78 (30.0)	103 (31.9)
Some clients	107 (40.8)	30 (56.6)	67 (26.2)	98 (30.8)	16 (29.6)	106 (40.8)	125 (38.7)
No clients	106 (40.5)	13 (24.5)	160 (62.5)	178 (56.0)	9 (16.7)	51 (19.6)	61 (18.9)

One third of respondents stated that condoms were used during oral sex with all clients; 69% and 59% of respondents reported that condoms were used during vaginal or anal sex respectively with all clients (Table 3). There was no difference in the proportion of male respondents reporting that condoms were used with all clients during oral sex (21%) compared to females (35%), *p* = 0.064. However, a higher proportion of men reported that condoms were used with all clients during anal sex (72%) compared to women (53%; *p* = 0.028). Female brothel workers were more likely to report that condoms were used with all clients for vaginal (80%), anal (71%), and oral (55%) sex compared to female workers who did not work in brothels (vaginal 64%, *p* = 0.010, anal 47%, *p* = 0.010, and oral sex 28%, *p* < 0.001; Table 3).

**Table 3:** The use of condoms with clients in an average week (by respondents reporting this type of sex with clients).

	**Vaginal sex**	**Anal sex**			**Oral sex**		
	**Women only** *n =* 241 ***n (*%)**	**Men** *n =* 46 ***n (*%)**	**Women** *n =* 148 ***n (*%)**	**All** *n =* 202 ***n (*%)**	**Men** *n =* 51 ***n (*%)**	**Women** *n =* 243 ***n (*%)**	**All** *n =* 303 ***n (*%)**
**ALL SEX WORKERS**							
All clients	165 (68.5)	33 (71.7)	79 (53.4)	119 (58.9)	11 (21.6)	86 (35.4)	100 (33.0)
Most clients	66 (27.4)	8 (17.4)	47 (31.8)	56 (27.7)	6 (11.8)	52 (21.4)	60 (19.8)
Some clients	9 (3.7)	4 (8.7)	4 (2.7)	8 (4.0)	21 (41.2)	61 (25.1)	84 (27.7)
No clients	1 (0.4)	1 (2.2)	18 (12.2)	19 (9.4)	13 (25.5)	44 (18.1)	59 (19.5)
**FEMALE BROTHEL WORKERS ONLY (*****n** =* **79)**							
	***n** =* **69**		***n** =* **38**			***n** =* **66**
All clients	55 (79.7)	–	27 (71.1)	–	–	36 (54.6)	–
Most clients	13 (18.8)	–	7 (18.4)	–	–	13 (19.7)	–
Some clients	1 (1.5)	–	3 (7.9)	–	–	11 (16.7)	–
No clients	0	–	1 (2.6)	–	–	6 (9.1)	–

There was no association between reported illicit drug use in the past 6 months and condomless sex with clients. However, those respondents who reported drinking six or more standard drinks on any one occasion at least weekly were less likely than other respondents to report condom use always during oral, vaginal and anal sex with clients (Table 4).

**Table 4:** Proportion of respondents reporting that all clients used condoms in an average week according to reported use of illicit drugs in the past 6 months and consuming six or more standard drinks on any one occasion at least weekly.

	**Always using condoms during vaginal sex (female respondents only)**		**Always using condoms during anal sex**		**Always using condoms during oral sex**	
**Illicit drug use in the last 6 months**	***n (*%) *n* = 241**	***p* value**	***n (*%) *n =* 202**	***p* value**	***n (*%) *n =* 303**	***p* value**
Yes	77 (69.4)	0.780	71 (68.4)	0.149	41 (27.9)	0.066
No	88 (67.7)		48 (53.3)		59 (37.8)
**Binge drinking at least weekly**^*a*^	***n (*****%)** ***n** =* **235**	***p*** **value**	***n (*****%)** ***n** =* **201**	***p*** **value**	***n (*****%)** ***n** =* **297**	***p*** **value**
Yes	40 (50.6)	< 0.001	37 (45.7)	0.001	19 (18.6)	< 0.001
No	120 (76.9)		82 (68.3)		79 (40.5)

a *Reported consuming six or more standard drinks on any one occasion at least weekly*.

A lower proportion of sex workers who reported getting safe sex information from clients reported condom use with all clients in an average week during all forms of penetrative sex compared to respondents who reported getting safe sex information from other sources (Table 5).

**Table 5:** Proportion of respondents reporting that all clients used condoms in an average week according to sources of information about safe sex skills (among respondents having this type of sex with clients).

	**Always using condoms during vaginal sex** ***n*** = **232**		**Always using condoms during anal sex** ***n*** = **129**		**Always using condoms during oral sex** ***n*** = **266**	
**Source of safe sex information**	***n (*%)**	***p* value**	***n (*%)**	***p* value**	***n (*%)**	***p* value**
Clients	26 (45.6)	< 0.001	18 (42.9)	0.014	11 (17.2)	0.004
Other sources	132 (75.4)		88 (64.2)		74 (36.6)

### In-depth interviews

We interviewed everyone who agreed to be interviewed, and undertook 17 in-depth interviews. Twelve of the interview participants were female at birth, two were trans-women and three were men. Experience in the sex work industry varied from 6 months to more than 20 years. Fourteen participants were currently sex workers, two were not currently working in the industry but had done so in the last 12 months, and one was transitioning out of the sex industry.

### Client requests for condomless sex

The majority of participants, particularly those who had worked in the industry for several years, indicated that the demand for condomless sex, particularly oral sex, had increased recently. Sex workers who worked in brothels as well as those who worked privately reported requests for condomless sex–in the brothel setting usually at the time of service, as described by this participant who works in a brothel.

I feel strongly against natural services, like you know people ask all the time pretty much every second client now when receiving like the standard service (ID03, woman)

More than one interview participant noted that decreased demand for sexual services ascribed to a downturn in the WA economy made it more difficult for sex workers to refuse to provide unprotected services.

…*that's now getting to the point where like 3 years ago protected services for full-service were pretty close to 100%, and there was a very high level of protected services for oral sex. And just because workers aren't in a position to pick and choose anymore, particularly for oral sex that's gone way, way down…. like workers who don't do those things are not getting repeat clients. And so it's just across the board there's a lot of pressure to–particularly with oral–start doing unprotected oral… Well, like there are very, very few people who, given the choice are going “not I'm not going to use a condom.” Probably no people. But it's just people have got rent to pay (ID14, woman)*

All stated that at least some of their clients requested condomless sex. For some, this was more occasional, and they felt able to screen out these clients during the initial phone call, who can then seek condomless sex elsewhere if they wish.

They'll usually try and do it [ask] on the phone because they know they can get someone who will (ID06, woman)

Those who received requests for condomless sex less frequently than others ascribed this to how they promote their services; by being clear about what services they do and do not provide. One participant has recently focused on creating her brand. She stated that after doing this, requests for condomless sex decreased.

I've got a website and done kind of like various things to get myself noticed online, and as I've done that and invested more and got more expensive, I don't know if that has any kind of correlation to it, I'm not entirely sure, but definitely the more effort I put into creating a brand presence, the less requests for natural services I started to have (ID05, woman)

A few also reported that they had seen advertisements from other sex workers offering unprotected oral sex. This was corroborated by our own findings. Some saw this as a threat to themselves and the industry by setting up client expectations of being able to purchase condomless sex and thereby making it more difficult for them to be able to make a living without offering it.

I think should have a better law enforcement to stop websites like… allowing advertisement for sex with no condom…It's very risky to all over Australia sex industry (survey respondent, free text)

Another participant expressed anger at people advertising condomless sex, even though she offered unprotected oral sex herself.

But now, everyone is fucking advertising that shit [unprotected oral sex] everywhere. I don't like that because I am funny old prude and like to keep shit behind closed doors. Like my ad, you couldn't read my ad and know that I do unprotected oral services sometimes (ID10, woman)

This was not universal however, and other participants were clear that they didn't mind what other workers did. They felt that people should be allowed to offer and provide the services that they wanted to.

The colloquial term for condomless sex is “natural services,” which is used by clients, sex workers and others in the sex industry. The term has connotations of increased desirability of condomless sex, and also may reduce clients' perceptions of the transactional nature of the sexual service they are purchasing. One participant described why she didn't like to use the term “natural services”

I have this thing for starters where I don't call it natural because that sounds like you're implying that is unnatural to have sex with a condom (ID10, woman)

### Strategies to decline condomless sex

A number of interview participants described how they refused to provide condomless sex, even with persistent clients. Some of those who worked in brothels currently or previously described being coached by other sex workers on how to refuse to provide a service that they didn't want to provide. They also described feeling supported in refusing by knowing that there were other people around in the brothel that they could call on if feeling threatened.

Yeah, and being surrounded by the right environment of women around me saying, do not feel pressured to perform anything if you do not want to do it… Don't feel pressured… I'm not going to do it. I'm not going to put my life and my sexual health in jeopardy for someone else for what $150? (ID17, woman)

So that's another thing about the team environment is knowing that you've got that support network of, if you do have a client who cracks the shits when you won't do natural, you've either got five, six girls all outside, plus your manager, your receptionist etc (ID08, woman)

Interview participants who worked privately also described their own strategies for declining condomless sex with persistent clients. A couple of participants described giving three warnings and then telling the client to leave if they continued to persist. Another couple of participants described being very clear up front of what services they provide and therefore being clear that there was no room for negotiation.

And they'll throw anything on once, but if they get into the room, and they push me, I give them three warnings. And if they say something, you know inappropriate or pushy, I'll be like I've already got the money in my hand mother fucker. I gave you three warnings. That's it. Thanks for finishing my job early for the day. So mentally I feel like I'm being bossy, but to announce that, I'm like, oh, I'm not going to let anybody push me around that I can say something to (ID06, woman)

Yeah, and then I, I actually, I have a lot of control, because I built it, this is my service. As time goes by I have actually fine-tuned it and I give them a list, like how you go to a hotel and you have a menu. And so, if you only have to order what you have on the menu, you can't just order what they don't have. And so, that week, I said this is all I will do. These are my limits. This is how far I will go. You take it or leave it (ID04, man)

### Participation in condomless sex with clients

A number of participants reported that they provided condomless sex at least some of the time (predominantly oral sex). A couple of these had worked in the sex industry for many years but had only recently offered this service. The increasing demand for condomless sex and the knowledge that potential clients could obtain that service elsewhere was an important motivator for some. One participant described providing more oral sex after offering condomless oral sex to clients.

Like probably 90% of people will ask for a natural service. I was so steadfast for the first two years. I was like fucking no, no, no, no. And now I do natural oral for shit now [low payment] because now I just like give up. There's just no fucking way to beat the system on that one because if I charged 700 bucks, and I gave a shit about my appearance, I'd probably get away with it but I can't be bothered (ID06, woman)

They used to occasionally ask for natural oral, but not that often. It's got to the point now where they expect that they can pay extra for it. Don't get me wrong, you can still make a living without doing it, however they ask about natural sex more often. They never even used to ask about that. And the other difference I noticed, because I started offering, started changing what I offer, and what I primarily sell at the moment, and I still do my standard full service and all of that jazz, but the primary thing I sell is a blow job service right (ID10, woman)

Related to the above was what one participant described as a sense of wanting to be upfront in offering condomless sex rather than changing her mind during the service in response to client demand. In this way the participant could maintain her sense of control of the situation and also ensure upfront payment for the additional service.

…*that's why I kind of decided to offer it, because if you don't decide to offer it, and then you feel obliged to do it in the middle of the booking, you feel like a fucking piece of shit afterwards because you feel like you got pushed into doing something you didn't want to do. And so I was like, well, I may as well make the choice to do it because half the fucking time I am doing it anyway. I was feeling shitty about doing it because I felt morally, or whatever, I was doing it; it didn't twist right in my head and I felt guilty as shit. Like I'd been tricked, whereas now I'm just like, man, whatever, you've just been tricked into an extra 50 bucks (ID06, woman)*

Several sex workers reported a reduction in the demand for sexual services in WA due to the downturn in the WA economy, and possibly an increase in the number of sex workers. The combination of the reduction in demand for services and the increasing demand for condomless sex was what led to the decision for a couple of participants to offer condomless sex. This is in addition to the ability to charge more for providing condomless sex.

That, but the thing was, why was I doing that [providing unprotected sex]? It was because it was just getting hard to make money (ID11, woman)

A participant who recently left WA with a view to exiting the industry said that she hadn't offered condomless sex but thought that she would have if she'd stayed in the industry.

If like, if I stayed in WA and stayed in the industry, I probably would have had to do it. You know, like it was the way things were going (ID14, woman)

A male sex worker who had always offered unprotected oral sex described now providing unprotected anal sex to some clients. He described feeling bad about doing this but felt that he had to do it sometimes as he could no longer compete with younger well-built men. Therefore, his decision was a financial one.

I felt stupid and dumb and desperate and I was in such a low point I was like, “Oh my god, I ring my mum for the millionth time asking for money.” Yes like what we will do bareback. And basically I felt shit about it (ID02, man)

This sense of competition with other workers was also described by some female sex workers in the context of knowing that they will potentially receive poor online reviews if they don't offer unprotected oral sex, or risk clients not returning.

There, a lot of the girls that do start in the industry are quite young, and they do get sucked into the whole review system by punters if you want to call them that, clients. They will review girls and then complain that they didn't offer this service. And they're charging so much money and they really should be offering this (ID17, woman)

### Risk management strategies

Some interview participants who offered or provided condomless sex described risk management strategies, such as inspection of the clients' genitals for evidence of herpes, discharge, ulceration or genital warts. One also noted that unprotected oral sex is less risky for HIV transmission and also for the male client, but is potentially more risky for the sex worker in terms of other STIs. Therefore, the onus is more on the sex worker than the client in terms of protecting themselves from STIs.

*You're very unlikely to pick anything up by sticking your dick in someone's mouth, but you're very likely to pick something up the other way from letting someone stick their dick in your mouth. So I guess I feel concerned that I am going to catch something. Like I do always check them, do a visual STI check, but obviously they are not always 100% because a lot of things don't show symptoms, etc…. And also, oral sex is quite low in terms of transmission. And most of the things you can catch that way are curable. Or if they aren't curable like warts or herpes you are more likely going to need to see something going on in order for it to infect you (ID10, woman)*.

A male respondent who said he did occasionally provide unprotected anal sex described two risk management strategies: being the insertive partner (mostly); and trusting his instincts that the client was safe.

…*but in this last year I have done it [unprotected anal sex] a few times with people that I had the instinct that they were safe and I didn't have any condoms. So, I did that. And it was me fucking them, also the other way, but maybe only twice. But yeah it is something that has happened and something I have always avoided apart from just this year (ID02, man)*.

## Discussion

We found higher rates of condomless sex with clients reported by sex workers in our study than we would have expected from previous studies. Condom use was lowest during oral sex with clients, with 33% of respondents reporting condom use with all clients during oral sex. Fifty nine percent of sex workers reporting having anal sex with clients reported condom use during anal sex with all clients, and 67% of female respondents reported condom use during vaginal sex with all clients. Female sex workers working exclusively in brothels reported higher rates of condom use compared to those working privately, with 55, 71, and 80% of respondents reporting condom use with all clients during oral sex, anal sex, and vaginal sex respectively. Increasing client demand for condomless sex and concerns about reductions in income if condomless sex is not offered were important factors relating to the provision of condomless sex.

Condomless sex with clients was not associated with drug use in our study but was associated with binge drinking at least weekly. Condomless sex was also associated with obtaining information about safe sex from clients rather than other sources such as sex worker organizations and health services, suggesting that a lack of engagement with education, health and support services may be an important factor in condomless sex. These data point to the importance of peer-based education and support services for sex workers as well as accessible health services for sex workers. This has become more challenging with the growth in private sex work, and points to the need for greater investment in outreach services.

Interview participants reported a range of strategies to refuse condomless sex including upfront promotion of which services they do provide, giving warnings that the service will not proceed if the client insists, and seeking support from workmates in the brothel setting. Several interviewees stressed the importance of knowing that they can refuse to provide a particular service, and that the support of peers was important for them in feeling confident about refusing to provide services (including condomless sex) that they did not wish to provide. These findings again stress the importance of peer support, and the need to ensure that sex workers working privately are also able to access this support.

We found much higher rates of condomless sex than was found in previous studies using the same survey instrument (1, 2). While the current and previous studies used the same questions in relation to condom use, the sampling methodologies were different. The 2007 studies recruited sex workers in brothels, who completed their questionnaires on-site. While we also approached sex workers in brothels and provided them with hard-copy surveys, the majority of our survey respondents completed the survey online and were recruited via social networks and social and other electronic media. In spite of these study differences, our finding of a reduction in condom use is likely to reflect a real change, given that it is consistent with our observations and reports from sex workers. Our findings are also consistent with findings from a study of attendees at a Sydney sexual health clinic undertaken in 2009–2011, which found that 75% of female sex workers reported inconsistent condom use during oral sex with clients (4). A recent study of online advertisements by private sex workers in Sydney, Australia, found that 50% of female private sex workers offered condomless oral sex (14). This is also consistent with our findings of low rates of consistent condom use during oral sex with clients. Studies conducted in other countries have also found lower rates of condom use with clients among private compared to brothel-based sex workers (15–17).

Oral sex, particularly for the insertive partner, is low risk for HIV transmission (18). Our findings of higher levels of reported condomless oral sex compared to anal and vaginal sex is likely to reflect an assessment of HIV risk in relation to condomless sex by sex workers and their clients. However, condomless oral sex can be important for the transmission of other STIs such as chlamydia and gonorrhea (19). In addition, pharyngeal gonorrhea could be an important reservoir for ongoing transmission (4, 20). While some interview participants described using visual inspection of a client's genitals as a risk management strategy for reducing the risk of acquiring a STI from their clients during condomless oral sex, visual inspection is ineffective at detecting all STIs. Our findings point to the importance of screening for pharyngeal gonorrhea and chlamydia when doing sexual health checks with sex workers.

A systematic review of structural drivers of HIV infection among sex workers found that strong client demand and economic incentives were drivers of inconsistent condom use with clients, consistent with our findings (17). The review also found that legislation making sex work illegal was a driver of inconsistent condom use through increasing stigma, economic uncertainty and workplace violence. A small proportion of brothels (18%) that we visited had signs in their waiting rooms indicating a requirement for clients to use condoms. This may reflect WA legislation where brothels are illegal (17). In their systematic review, Shannon et al. also found that venue-based policies on condom use were associated with a reduction in condomless sex between sex workers and clients (17). Making these policies explicit through signage and making condoms available on-site, therefore, is likely to assist sex workers to refuse condomless sex and may also reduce the number of clients requesting unprotected services. The findings of this review, together with our own, demonstrate the importance of decriminalization of sex work in improving the health and safety of sex workers in WA.

Our findings suggest that client demand for condomless sex is a strong driver of the reduction in consistent condom use among sex workers in WA. We did not interview clients so were unable to ascertain what is behind this increasing demand. Many men and women find sex without condoms to be more pleasurable than sex with condoms (21). However, as the demand for condomless sex appears to be increasing, there are likely to be other factors also contributing to this, including possibly a perception by clients that condomless sex is not risky. Further research is needed in order to gain a deeper understanding about what is leading to an increasing demand for condomless sex by clients in WA in order to inform interventions to reduce this demand and support sex workers in refusing to provide condomless sexual services should they wish to do so.

There were a number of limitations in our study. While our study was relatively large, the sample was not randomly selected and our recruitment strategies relied heavily on word of mouth, and social and other electronic media. While our sample may therefore not be representative of all sex workers in WA, our sample was quite diverse, and we surveyed a high proportion of private sex workers, reflecting the current sex industry. We also did not obtain samples for testing for STIs. However, there are recent Australian data available in relation to STIs among sex workers, which shows an incidence of STIs among sex workers that is increasing, but comparable to the general population (22).

Finally, our questionnaire did not attempt to quantify the proportion of clients who used condoms, rather using terms such as “most” or “some,” which may be interpreted differently by different respondents. For consistency our questions about condom use were the same as those used in an earlier study in WA (2), which also used these terms rather than trying to quantify condom use.

## Conclusions

We found unexpectedly high levels of self-reported inconsistent condom use among male and female sex workers in WA during all forms of penetrative sex with clients. This was higher than was found in a study undertaken a decade ago. We also found high levels of reported demand for condomless sex from clients, and that the lower condom use does not appear to be driven by sex workers themselves. Interventions to increase condom use during sex work need to be informed by an understanding of the drivers of the demand for condomless sex by clients of sex workers and further research in this area may be justified. Increasing the level of peer-based support and education for sex workers, particularly private sex workers, may contribute to increased use of condoms with clients, together with targeted education for clients, as informed by further research. Finally, decriminalization of sex work in WA is recommended in order to facilitate peer outreach, reduce stigma, and allow brothels to more openly promote condom use.

## Author contributions

LS lead the overall study, analyzed the data, and wrote the manuscript. JH, KM, JB, BD and RL contributed to data collection and interpretation. All authors read and approved the final manuscript.

### Conflict of interest statement

The authors declare that the research was conducted in the absence of any commercial or financial relationships that could be construed as a potential conflict of interest.
